# Lymph Node Ratio as a Prognostic Factor for Oral Tongue Squamous Cell Carcinoma: A Retrospective Study

**DOI:** 10.7759/cureus.44109

**Published:** 2023-08-25

**Authors:** Gidean A Sundaram, Jerry Joe Chokkattu, Murugesan Krishnan, Santhosh P Kumar, Senthilmurugan M, Saravanan Lakshmanan

**Affiliations:** 1 Oral and Maxillofacial Surgery, Saveetha Dental College and Hospitals, Chennai, IND; 2 Prosthodontics, Saveetha Dental College and Hospitals, Chennai, IND

**Keywords:** extra nodal extension, lymph node ratio, oral cavity cancer, prognostic factors, oral cavity squamous cell carcinoma, survival

## Abstract

Background

Oral squamous cell carcinoma (OSCC) incidence and its mortality have increased recently. The oral part of the tongue is one of the commonest sites for OSCC. Apart from Tumour-Node-Metastasis (TNM) staging, lymph node ratio (LNR) has been implicated as one of the useful predictors for the better clinical outcome of the disease. The aim of the present study was to assess the LNR as a prognostic factor for patients having oral tongue squamous cell carcinoma (OTSCC).

Materials and methods

It is a retrospective study of 122 patients with OTSCC who were managed primarily by surgery with curative intent from January 2014 to December 2016. The mean lymph node ratio was measured and compared with various parameters of clinical outcome such as five-year overall survival (OS), five-year disease-free survival (DFS), locoregional failure (LRF) within three years, and distant metastasis (DM) within five years using Kruskal-Wallis Test followed by Mann-Whitney Post Hoc Test. The association of LNR with other tumor characteristic features like perineural invasion, extra-nodal extension (ENE), and histopathological grading was also elicited.

Results

The study population's mean age was 50.5 ± 11.77 years. Among them, 85 were males and 37 were females. On comparing the mean LNR value with patient status after primary treatment, the patients with minimal LNR value had statistically significant five-year OS and five-year DFS (p< 0.001). High mean LNR values were associated with other adverse features like perineural invasion and ENE, which were statistically significant (p<0.001). Receiver operator characteristics (ROC) curve analysis for the LNR parameter for determining the cut-off (0.02) between OS and DFS had 86% sensitivity and 40% specificity.

Conclusion

The LNR could be an important prognosis factor for OTSCC that helps in determining better clinical outcomes.

## Introduction

Oral cancer accounts for 30% of head and neck cancers, out of which 95% are squamous cell carcinoma [1]. The incidence of anterior 2/3rd tongue (oral part of the tongue) cancer is the increasing trend in recent years [2]. Approximately 405,000 new oral cancer cases are diagnosed each year and above 50% are oral tongue cancers. The most common site is along the lateral border and ventral surface of the tongue [3]. In oral cancers especially in the tongue, the incidence of lymph node metastasis (N+) is high. Occult metastasis might be present even in the clinically negative neck (N0). Nearly 20-34% of cases present with clinically positive lymphadenopathy [4]. Neck metastasis is influenced by various factors like size, stage, tumor thickness, primary neoplasm location, and the presence of perineural invasion [5]. Lymph node metastasis is considered a well-known risk factor for poor clinical outcomes and locoregional recurrence.

Tumour-Node-Metastasis (TNM) classification is considered a very important prognostic factor after resection, and pathological TNM staging (pTNM) contains a relevant impact on the prognosis and survival of the patient [6]. In addition to pTNM, lymph node ratio (LNR) also has relevance in the prognosis of head and neck cancers. It is calculated as the ratio of tumor-positive lymph nodes to the number of dissected lymph nodes. LNR has been implicated as one of the useful predictors for the better clinical outcome of the disease in patients with oral tongue squamous cell carcinoma (OTSCC) [7]. The aim of the present study was to assess the LNR as a prognostic factor for patients having OTSCC.

## Materials and methods

This was a single institutional retrospective study. 122 subjects having biopsy-proven oral squamous cell carcinoma (OSCC) and treated primarily by surgery with curative intent from January 2014 to December 2016 were included in this study. The peripheral margin of 1-1.5 cm was included in the surgical resection all around the tumor and modified radical neck dissection (MRND) III was executed in all the cases. All tissue samples obtained during surgery were sent for histopathological examination. Tumor size, lymph node metastases, vascular invasion, perineural invasion, TNM staging, and such pathological information were collected. Based on the histological differentiation of tumor cells, it was categorized by experienced pathologists [8]. In the harvested cervical lymph nodes further detailed examination of the size (greatest dimension) of the largest metastatic focus in the lymph node, and extranodal extension (invasion of tumor cells beyond the capsule of lymph nodes) were analyzed by experienced pathologists. Perineural invasion was diagnosed with the histopathological sections where tumor cells involve any of three nerve sheath layers or tumor adjacent to more than 1/3rd of the nerve circumference. Patients received adjuvant radiotherapy (RT) and chemotherapy (CT) based on the adverse features present in the postoperative histopathological report.

In the present study, OTSCC was documented according to the American Joint Committee on Cancer TNM staging classification [9]. Patients with T4a and T4b disease were not included because T4a oral tongue disease almost always presents with extensions involving the pharyngeal part of the tongue. Likewise, patients with T4b presented with an inoperable stage and were excluded from the study. Patients with N3 nodal disease were also excluded because patients with this stage usually present with large nodes fixed to the skin and underlying structure and/or with surface ulceration and are usually in the inoperable stage. The subclassification of N2 nodal disease is omitted and taken as a single stage because it doesn't have any significant role in calculating the LNR ratio [10]. Patients with positive margins and patients who have undergone neoadjuvant chemotherapy were also excluded to avoid bias in the study results. 

Follow up schedule in the study population was once a month for the first three months, from the fourth month every three months once up to one year of the follow up, and yearly follow-up for up to five years. If follow-up records were incomplete, the patient was excluded from the study. The mean lymph node ratio was calculated as the ratio of tumor-positive lymph nodes to the number of dissected lymph nodes. It was compared to various parameters of clinical outcome such as five-year overall survival (OS), five-year disease-free survival (DFS), locoregional failure (LRF) within three years, and distant metastasis (DM) within five years.

Statistical analysis was done using Statistical Package for Social Sciences [SPSS] (Windows Version 22.0 Released in 2013. Armonk, NY: IBM Corp.,). In descriptive statistics, frequency and proportions were utilized, whereas mean and standard deviation (SD) were employed for continuous data. Kruskal-Wallis Test followed by the Mann-Whitney Post Hoc test was used to compare the mean LNR values based on the patient status after primary treatment. Mann-Whitney / Kruskal-Wallis test was used to compare the mean LNR values based on tumor characteristics. The chi-square test was used to compare the sociodemographic and tumor characteristics based on OS and DFS among the study patients. Receiver operator characteristics (ROC) curve analysis was done for the LNR parameter for defining the cut-off between OS and DFS. The statistical level of significance was kept at P<0.05.

## Results

**Figure 1:**
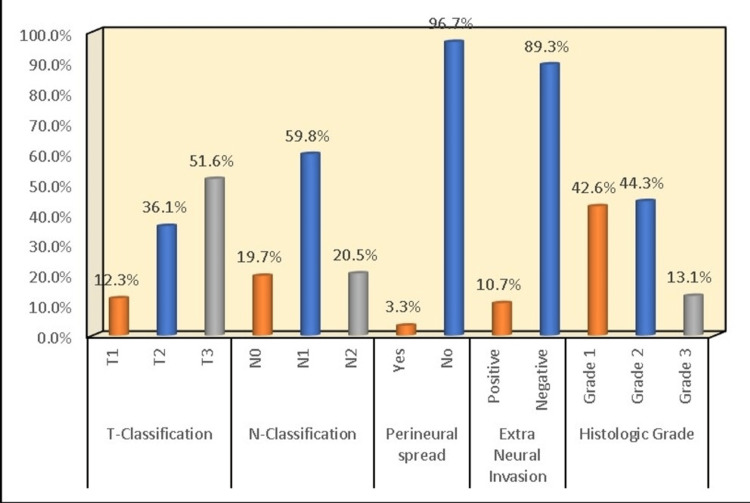
Distribution of tumor characteristics among the study population

The age of 122 patients in this study ranged from 21 to 71 years, with an average age of 50.5 ± 11.77 years. Among them, 85 were males, and 37 were females (Table 1). Based on tumor size, 15 patients with T1 disease, 44 patients with T2 disease, and 63 patients with T3 disease were identified in our study. According to the cervical node metastasis, 24 patients had N0 neck, and 73 and 25 were reported with N1 and N2 nodal disease, respectively. Perineural invasion (PNI) was present in four patients and extranodal extension (ENE) was present in 13 patients. Recent National Comprehensive Cancer Network (NCCN) guidelines have given a grading system based on Border's criteria [8,11]. In our study, we have followed the same grading system. Histopathological examination was done by two individual pathologists. Grade 1 (Well differentiated) category was given to the tumors having a very close histological and cytological resemblance to the oral cavity mucosa. Different proportions of basal and squamous epithelial cells with intercellular bridges were noticed. These tumor cells have shown keratinization along with minimal nuclear and cellular pleomorphism. Grade 2 (Moderately differentiated) category was given to the tumors having intermediate histological and cytological features between well and poorly-differentiated tumors. Grade 3 (Poorly differentiated) category was given to the tumors having barely any histological or cytological resemblance to the oral cavity mucosa. Frequent mitotic activity, atypical mitosis, and cellular and nuclear pleomorphism are the typical features of this category of tumors. 52 patients with well-differentiated squamous cell carcinoma (Grade 1), 54 patients having moderately differentiated squamous cell carcinoma (Grade 2), and 16 patients having poorly differentiated squamous cell carcinoma (Grade 3) were noticed in this study (Table 2 and Figure 1).

**Table 1:** Age and gender distribution among the study population

Variable	Category	N	Percentage
Age (in years)	21-30	4	3.3%
	31-40	19	15.6%
	41-50	38	31.1%
	51-60	38	31.1%
	61-70	17	13.9%
	> 71	6	4.9%
Gender	Males	85	69.7%
	Females	37	30.3%

**Table 2:** Distribution of tumor characteristics among the study population Grade 1 - Well differentiated, Grade 2- Moderately differentiated, Grade 3 - Poorly differentiated oral tongue squamous cell carcinoma

Variable	Category	N	Percentage
T-Classification	T1	15	12.3%
	T2	44	36.1%
	T3	63	51.6%
N-Classification	N0	24	19.7%
	N1	73	59.8%
	N2	25	20.5%
Perineural invasion	Yes	4	3.3%
	No	118	96.7%
Extra nodal extension	Positive	13	10.7%
	Negative	109	89.3%
Histologic grade	Grade 1	52	42.6%
	Grade 2	54	44.3%
	Grade 3	16	13.1%

The patients below the average age of 50.5 years were considered younger patients in our study. Among the five-year OS, the younger patients were 60% and among the five-year DFS, the younger patients were 46.5 %. Among the five-year DFS males were 58% and among the five-year OS there were no females. In five-year OS, 20% had T1 disease, 40% had T2, and 40% had T3 disease. In five-year DFS T1, T2, and T3 were 28%, 44%, and 28%, respectively. In five years, OS patients with N0 nodal involvement were 20% and N1 were 80%. In five-year DFS, N0, N1, and N2 were 35%, 51%, and 14%, respectively. In five-year OS and five-year DFS, patients with PNI were nil. In five-year OS and five-year DFS patients without extranodal extension (ENE -ve) were 93% and 95%, respectively. Among five-year OS, patients with Grade 1 histological features were 53%, and Grade 2 and Grade 3 were 26% and 20% respectively. Likewise, five-year DFS Grades 1, 2, and 3 were 49%, 42%, and 9%, respectively (Table 3).

**Table 3:** Comparison of sociodemographic and tumor characteristics based on overall and disease-free survival OS: overall survival; DFS: disease free survival; * statistically significant (chi-square test)

Variable	Category	OS		DFS		P-Value
		N	Percentage	N	Percentage	
Age (in years)	Younger (< 50.5)	9	60.0%	20	46.5%	0.30
	Older (>50.5)	6	40.0%	23	53.5%	
Gender	Males	15	100.0%	25	58.1%	0.003*
	Females	0	0.0%	18	41.9%	
Tumor	T1	3	20.0%	12	27.9%	0.66
	T2	6	40.0%	19	44.2%	
	T3	6	40.0%	12	27.9%	
Nodal status	N0	3	20.0%	15	34.9%	0.11
	N1	12	80.0%	22	51.2%	
	N2	0	0.0%	6	14.0%	
Perineural invasion	Yes	0	0.0%	0	0.0%	..
	No	15	100.0%	43	100.0%	
Extra nodal extension	Positive	1	6.7%	2	4.7%	0.77
	Negative	14	93.3%	41	95.3%	
Histological grade	Grade 1	8	53.3%	21	48.8%	0.41
	Grade 2	4	26.7%	18	41.9%	
	Grade 3	3	20.0%	4	9.3%	
Lymph node ratio cut-off	≤ 0.02	12	80.0%	37	86.0%	0.55
	> 0.02	3	20.0%	6	14.0%	

On comparing the mean LNR value with patient status after primary treatment, the patients with minimal LNR value had better five-year OS and five-year DFS (p< 0.001) (Table 4). On comparing the mean LNR values with different statuses of the patients after primary treatment, it was statistically significant between five-year OS and locoregional failure within 3 years (p = 0.04). During the comparison of five-year OS and patients with distant metastasis within five years, it was found to be statistically significant (p = 0.04). Whereas mean LNR values compared between five-year OS and five-year DFS were not statistically significant. The mean LNR values compared between LRF within three years and DM within five years were also not statistically significant (Table 5). In five-year OS patients with LNR cut-off values ≤ 0.02 were 80% and LNR cut-off values >0.02 were 20%. In five-year DFS patients with LNR cut-off value ≤ 0.02 were 86% and LNR cut-off value > 0.02 were 14% (statistically borderline significance, p=0.55) (Table 3).

**Table 4:** Comparison of mean lymph node ratio OS: overall survival; LRF: locoregional failure; DM: distant metastasis; DFS: disease-free survival; * statistically significant (Kruskal-Wallis Test)

Status	N	Mean	SD	Min	Max	P-Value
5 Yrs. OS	15	0.018	0.028	0	0.07	<0.001*
LRF < 3yrs	44	0.042	0.037	0	0.12	
DM < 5 Yrs.	20	0.071	0.067	0	0.15	
5 Yrs. DFS	43	0.010	0.026	0	0.09	

**Table 5:** Multiple comparisons of mean difference in the lymph node ratio values between different statuses S1: 5 years overall survival; S2: locoregional failure < 3 years; S3: distant metastasis < 5 years; S4: 5 years disease free survival; * statistically significant (Mann-Whitney post hoc test)

Status	S1 vs S2	S1 vs S3	S1 vs S4	S2 vs S3	S2 vs S4	S3 vs S4
P-Value	0.04*	0.04*	0.08	0.14	<0.001*	<0.001*

**Figure 2:**
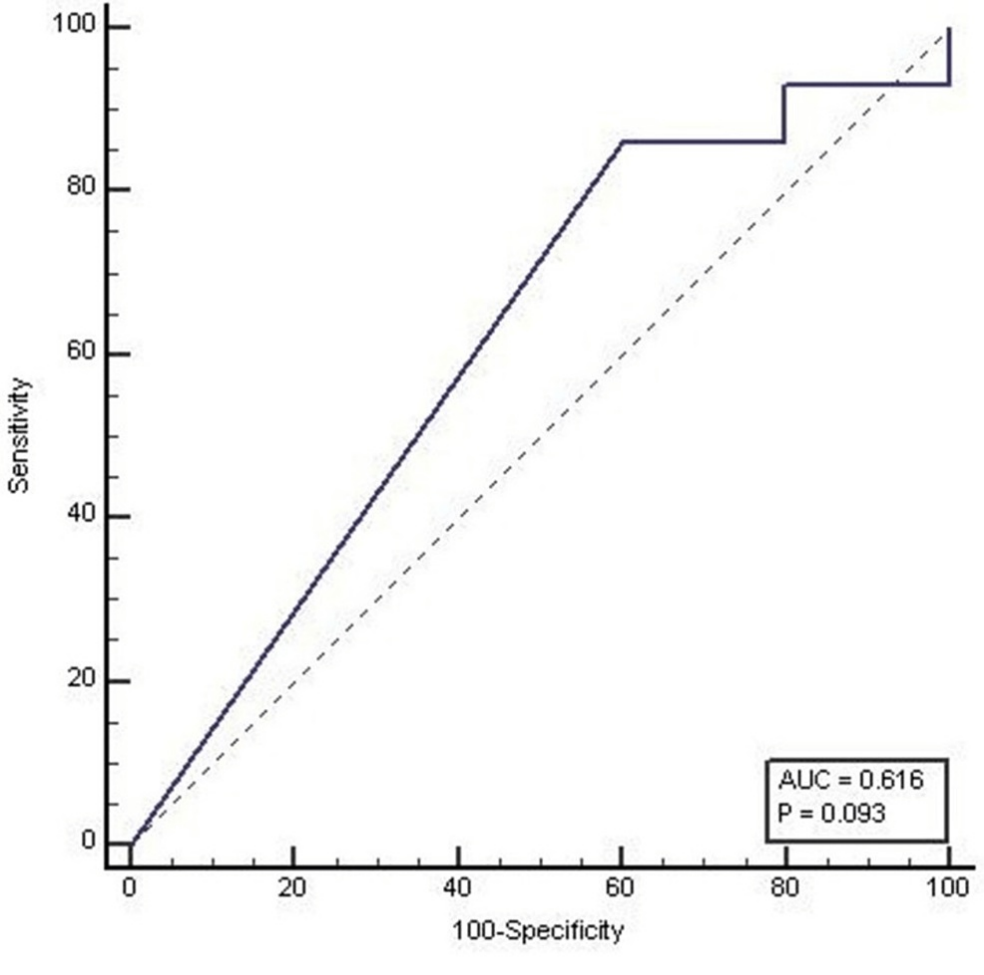
ROC curve analysis for the lymph node ratio parameter for defining the cut-off between overall survival and disease-free survival AUC: area under curve; ROC: receiver operator characteristics

High mean LNR values were also associated with other adverse features like perineural invasion (p<0.001), extranodal extension (p<0.001), and histological grading (Table 6). ROC curve analysis done for the LNR parameter for determining the cut-off (0.02) between OS and DFS had 86% sensitivity and 40% specificity (Table 7, Figure 2).

**Table 6:** Comparison of mean lymph node ratio values based on tumor characteristics * statistically significant (^a^ Mann-Whitney Test; ^b^ Kruskal-Wallis Test)

Variable	Category	N	Mean	SD	P-Value
Perineural invasion	Yes	4	0.110	0.000	<0.001*^a^
	No	118	0.030	0.043	
Extra nodal extension	Positive	13	0.074	0.030	<0.001*^a^
	Negative	109	0.027	0.043	
Histologic Grade	Grade 1	52	0.025	0.043	0.16^b^
	Grade 2	54	0.037	0.047	
	Grade 3	16	0.039	0.038	

**Table 7:** ROC curve analysis for lymph node ratio parameter for determining the cut-off between overall and disease-free survival status AUC: Area Under Curve; Sn: Sensitivity; Sp: Specificity; ROC: receiver operator characteristics

Variable	AUC	Std. Error	95% Confidence Interval		P-Value	Cut off	Sn (%)	Sp (%)
			Lower	Upper				
Lymph node ratio	0.62	0.07	0.48	0.74	0.09	0.02	86.05	40.00

## Discussion

OTSCC is more common in older males [12]. Tongue malignancy is usually not associated with traditional risk factors described for other oral cancer subsites [13]. Even though recent studies suggest that there is an upsurge in the incidence of tongue cancer among young adults and females [14], our study shows tongue cancer is more common in males. In our study, five-year OS was good among the younger patients than older ones as depicted in other studies [14], and not much difference in five-year DFS. However, other studies have also shown contradictory results, that younger patients will have more aggressive diseases and poor clinical outcomes when compared to older patients [15]. Most of the studies have shown that gender doesn't play a role in prognosis [14,16]. According to Horonto et al., hard palate tumors and distant metastases were the independent prognostic factors for poorer survival in females, and among males tobacco use history was considered as an independent prognostic factor for their poorer survival. However, regional metastasis was found to be a poor prognostic predictor in OSCC for both sexes [17]. Angélique Girod et al. has stated that the prognosis of OSCC was favorable in younger as well as older females [18]. In our study, slight male prediction in five-year DFS and five-year OS was noted, and the exact reason is not known.

The TNM stage has been proven in many studies as the most important prognostic factor and provides a reliable basis for treatment planning [19]. Even though it was well known that the lower T stage will have a better prognosis, in some cases advanced-stage diseases have a sluggish tendency to involve regional lymph nodes and some lower-stage diseases (T1 and T2) depict early and aggressive regional metastasis [20]. In our study it was noted that five-year OS was good among T2 and T3 diseases and five-year DFS was good in T2 disease. Likewise, five-year OS and five-year DFS were good in N2 disease and poor in N3 disease. This indicates that beyond the clinical dimension and nodal stage of the tumor, various molecular-level factors like p 53 mutations, angiogenesis-related factors, cyclin D1, cytokeratin 8/18, epidermal growth factor and transforming growth factor beta are also involved in determining the prognosis and clinical outcome of the disease [21].

Perineural invasion (PNI) is one of the well-documented prognostic predictors in OSCC and also indicates the aggressiveness of the tumor. In recent years, it has been discovered that tumors exhibiting PNI express a number of molecular factors, including Nerve Growth Factor (NGF)/tyrosine kinase A (TrkA), Neural Cell Adhesion Molecule (N-CAM), ICAM-5 (telencephalin), Claudin 1, Claudin 4, Laminin 5 (Laminin-332), Activin A, Bim/Bod, BAG-1, p73 and Snail [22]. In our study, none of the patients with PNI had a five-year OS or DFS. Extracapsular spread (ECS), also known as extranodal extension or lymph node extracapsular extension, is recognized as a significant determinant of tumor aggressiveness. Bhattacharya P and Mukherjee R documented that ECS adversely impacts locoregional failure and aggression [23]. Mair et al. reported 82.1% without nodal metastasis, 63% nodal metastasis without ECS, and 57.5% nodal metastasis with ECS among the 3-year DFS patients [24]. Our study depicted a similar pattern with five-year OS and five-year DFS among ENE-positive patients.

Histological staging is considered an independent prognostic factor in OSCC. Even early-stage disease behaves more aggressively if the histological grades are high [25]. In our study, five-year OS and five-year DFS were good in Grade 1 lesions and poor in Grade 3 lesions. There were extensive studies on LNR as an independent prognostic factor for various cancers such as colorectal, gastric, esophageal, and bladder [26-27]. LNR plays an important role in regional recurrence, distant metastasis, and ultimately the prognosis of the disease [28]. In our study, it was noted that mean LNR values are high for patients with locoregional failure within 3 years and distant metastasis within 5 years when compared with mean LNR values of patients with five-year OS and five-year DFS. But there was no significant difference in mean LNR values of patients with five-year OS and five-year DFS.

Feng Z et al. found that high LNR strongly correlated with advanced T and N stages, the severity of pathological grading, and ECS [29]. Liebig et al. have stated a significant correlation between PNI and tumors with regional metastases in pancreatic adenocarcinoma [30]. Even though PNI, ENE, histological grade, and LNR can act as independent prognostic factors, in our study it was interesting to note that high LNR values were associated with the above-mentioned prognostic factors, and the molecular pathogenesis for this association was not clear.

The limitation of this study is its retrospective nature, Human papilloma virus (HPV)-induced carcinoma tongue not being included, and the fact that a fixed cut-off value for LNR has not been established. Another limitation of the study is that the molecular factors responsible for the prognosis were not evaluated. To establish LNR as a prognostic factor, multi-institutional prospective studies using large numbers of samples are required.

## Conclusions

This study validates that LNR is an important prognostic factor in OTSCC independent of N stage disease, which is similar to other subsites of the oral cavity. LNR plays an important role in regional recurrence and distant metastases. LNR is also associated with other adverse features that favor negative clinical outcomes such as PNI and ECS.
